# Enhancement of Anticorrosive Performance of Cardanol Based Polyurethane Coatings by Incorporating Magnetic Hydroxyapatite Nanoparticles

**DOI:** 10.3390/ma15062308

**Published:** 2022-03-20

**Authors:** Afzal Haq Asif, Mahendra S. Mahajan, Nagaraja Sreeharsha, Vikas V. Gite, Bandar E. Al-Dhubiab, Feroze Kaliyadan, Shivakumar H. Nanjappa, Girish Meravanige, Dalal Mishary Aleyadhy

**Affiliations:** 1 Department of Pharmacy Practice, College of Clinical Pharmacy, King Faisal University, Al-Ahsa 31982, Saudi Arabia; ayase.ita@gmail.com; 2 Department of Polymer Chemistry, School of Chemical Sciences, Kavayitri Bahinabai Chaudhari North Maharashtra University, Jalgaon 425001, MS, India; vikasgite123@gmail.com; 3 Department of Pharmaceutical Sciences, College of Clinical Pharmacy, King Faisal University, Al-Ahsa 31982, Saudi Arabia; sharsha@kfu.edu.sa (N.S.); baldhubiab@kfu.edu.sa (B.E.A.-D.); 4 Department of Pharmaceutics, Vidya Siri College of Pharmacy, Off Sarjapura Road, Bangalore 560035, India; 5 Department of Dermatology, College of Medicine, King Faisal University, Al-Ahsa 31982, Saudi Arabia; ferozkal@hotmail.com; 6 Department of Pharmaceutics, KLE College of Pharmacy, Bangalore 560010, India; shivakumarhn@yahoo.co.in; 7 Department of Biomedical Sciences, College of Medicine, King Faisal University, Al-Ahsa 31982, Saudi Arabia; gmeravanige@kfu.edu.sa

**Keywords:** cardanol, hydroxyapatite, nanocomposite, anticorrosive coatings, polyurethane, renewable materials

## Abstract

The present investigation demonstrates renewable cardanol-based polyol for the formulation of nanocomposite polyurethane (PU) coatings. The functional and structural features of cardanol polyol and nanoparticles were studied using FT-IR and 1H NMR spectroscopic techniques. The magnetic hydroxyapatite nanoparticles (MHAPs) were dispersed 1–5% in PU formulations to develop nanocomposite anticorrosive coatings. An increase in the strength of MHAP increased the anticorrosive performance as examined by immersion and electrochemical methods. The nanocomposite PU coatings showed good coating properties, viz., gloss, pencil hardness, flexibility, cross-cut adhesion, and chemical resistance. Additionally, the coatings were also studied for surface morphology, wetting, and thermal properties by scanning electron microscope (SEM), contact angle, and thermogravimetric analysis (TGA), respectively. The hydrophobic nature of PU coatings increased by the addition of MHAP, and an optimum result (105°) was observed in 3% loading. The developed coatings revealed its hydrophobic nature with excellent anticorrosive performance.

## 1. Introduction

Corrosion is a process in which metallic objects become damaged, which affects various sectors of industries such as automobile, construction, aerospace, marines, and healthcare. Metal corrosion is one of the big problems that result in massive losses to the economy as well as human health [1,2,3]. In the case of biomaterials, corrosion prevention is crucial particularly to overcome inflammation and allergic reactions caused by biomaterials as these materials are constantly challenged by biological fluids [4]. Anticorrosive paints and coatings based on synthetic polymeric resins are used to minimize such types of losses and to improve the life span of metallic items [5,6,7]. The majority of polymeric resins used in the present paints and coatings are of petroleum origin. The depletion of petroleum sources and fluctuations in their prices made it necessary to find out some new alternatives such as renewable, readily available, and ecofriendly polymeric materials [8,9]. Renewable materials including carbohydrates, lipids, vegetable oils, cardanol [10], eugenol [11], etc., have been explored to use in the preparation of monomers and polymers such as alkyds [5], polyurethanes [6], polyesters [6], phenol-formaldehyde resins, epoxy [12], benzoxazines, etc. [13]. The resultant renewable source-based polymers have found applications in the different fields such as coatings, composites, microencapsulation, reactive diluents, foams, and so on. Among all renewable materials, cardanol has fascinated the researchers due to its unique chemical structure and possible chemical modifications through the availability of reactive sites such as phenolic hydroxyl group, aromatic ring, and long aliphatic carbon chain with unsaturation [13,14]. Aromatic ring provides chemical and thermal resistances, while the hydroxyl group provides good adhesion. A long aliphatic carbon chain provides good flexibility, excellent water resistance, and anti-corrosive properties [5]. 

Over the past decades, nanocomposite polymer coatings have been used to improve the corrosion, thermal, and mechanical properties of coating formulations [15,16]. They are developed from nano-sized fillers and polymeric resins. The polymeric resin helps to hold the nanofiller in the matrix that works as a reinforcing material or improves physicomechanical properties of the composites [17,18]. Various types of nano-size fillers such as SiO_2_ [19], ZnO [20], TiO_2_ [21,22], Fe_2_O_3_ [23], Fe_3_O_4_ [24], ZrO_2_ [25], Al_2_O_3_ [26], V_2_O_5_ [27], graphene [28], BaZrO_3_ [29], and other materials [30] have been used in coating matrix for desired applications such as the improvement of adhesion, gloss, corrosion, scratch, thermal, and scratch/wear resistance. The main purpose of nanoparticle incorporation in the coating formulation is to enhance physicochemical as well as corrosion resistance. The use of nanocomposites offer excellent barrier resistance, flame retardancy, and change in optical and electrical properties [31]. Additionally, the use of corrosion inhibitor is also possible based on precursors used in the formulation of coatings, thickness, and adhesion towards the metal surface [32]. The protective organic/inorganic hybrid composite coatings are prepared by the addition of magnetic hydroxyapatite nanoparticle (MHAP) as a reinforcing agent. The presence of MHAP has enhanced coating properties such as chemical, wetting, and corrosion resistances. Previous reports are available on the use of hydroxyapatite (HAP) for bone tissue engineering, controlled drug delivery, and as filler for composites coatings. Even HAP modified with silver was used in the formulation of antibacterial composite coatings [33] as bone substitutes [34], drug and protein carriers [35], dental implants [36], etc. Additionally, magnetically modified HAP was utilized in the removal of heavy metals such as uranium (VI) [37], lead ion [38], cadmium (II) [39], copper, nickel [40], and other pollutants for the water [41]. As per the literature, until today, MHAP has not been used in the designing of anticorrosive coatings for metal protection. 

The present study explored the potential of cardanol and its derivative as an environmentally friendly alternative for petroleum raw materials for applications in polymer resin and coating industries. In the present study, cardanol-based Mannich polyol has been used to synthesize renewable PU. Structural features of the prepared resin were confirmed by end group analysis as well as by spectroscopic methods. Simultaneously, magnetic hydroxyapatite nanoparticles were prepared and modified for their magnetic properties to improve the anti-corrosion properties of PU coatings upon mixing with polyol and further treatment with hexamethylene diisocyanate. The prepared PU composite coatings were tested for their physicochemical and anticorrosive properties.

## 2. Materials and Methods

Cardanol was provided as a gift sample by the Polymer Division of Atul Ltd., India. Hexamethylene diisocyanate (99%) (HDI), diethanolamine, (98%), and dibutyltin dilaurate (95%) (DBTDL) were purchased from Sigma-Aldrich, India. Ferrous chloride tetrahydrate, ferric chloride, potassium hydroxide, calcium nitrate tetrahydrate, diammonium hydrogen phosphate, and xylene were purchased from Loba Chemie, India. All other chemicals were used as received without any purification.

### 2.1. Synthesis of Cardanol Based Mannich polyol

**Scheme 1:**
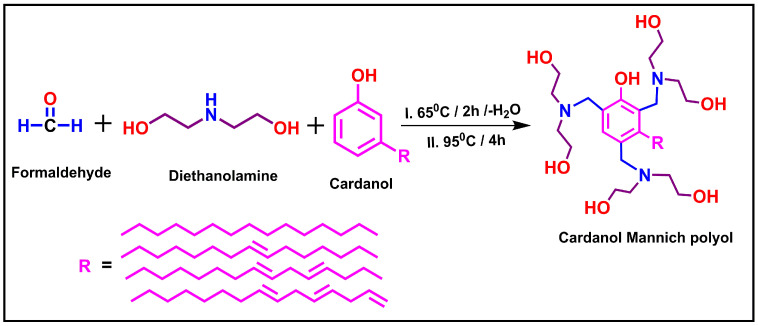
Synthesis of cardanol Mannich polyol.

The synthesis of cardanol Mannich polyol (CMP) was based on our previously reported method [42]. In the first step, the formation of oxazolidine was performed by reacting diethanolamine (0.2 M) with formaldehyde (0.2 M) in a three-necked flask equipped with a magnetic stirrer, condenser, and thermometer. The reaction mixture was heated at 65 °C for 2 h followed by distilling water off reaction to form oxazolidine intermediate. Thereafter, cardanol (0.066 M) was added dropwise in the reaction mixture for 30 min and the reaction was maintained to 95 °C for 4 h. The progress of the reaction was checked by conducting thin layer chromatography (TLC) in an ethyl acetate: hexane (20:80) system. Finally, the deep reddish-colored liquid of cardanol polyol was formed. The reaction for the synthesis of CMP is given in Scheme 1.

### 2.2. Synthesis of Magnetic Hydroxyapatite Nanoparticles

Magnetic hydroxyapatite nanoparticles were synthesized according to the literature procedure with some modifications [43,44]. In the typical process, ferrous chloride tetrahydrate (1.85 mM) and ferric chloride (3.78 mM) were dissolved in 30 mL DI water taken in a 250 mL round bottom flask under stirring at 500 rpm in the presence of nitrogen atmosphere. After 1 h, the completely clear orange color solution was formed. Then, 10 mL 30% ammonia solution was added dropwise into the reaction mixture and kept at 70 °C temperature for 1 h to form black color iron oxide nanoparticles. Entire synthesized iron oxide nanoparticles were well dispersed in a solution of calcium nitrate tetrahydrate (33.7 mM) and diammonium hydrogen phosphate (20 mM) prepared in a 250 mL beaker containing 50 mL DI water. Afterward, the pH of the solution was adjusted to 11 using ammonia solution. This mixture was stirred for 3 h at 90 °C temperature under a nitrogen atmosphere. Milky white particles formed were centrifuged and washed with water and ethanol to remove impurities. The resulting particles were magnetically separated from the medium using a local magnet and dried in an oven at 70 °C. Then, the particles were ground in a mortar pestle and filtered using 150 mesh size sieved for their uniform size and utilized for further applications.

### 2.3. Formulation of Polyurethane Nanocomposite Coatings

Mild steel (MS) panels were used as the substrate for the application of polyurethane (PU) nanocomposites coatings. The elemental composition of mild steel used is provided in Table 1.

**Scheme 2:**
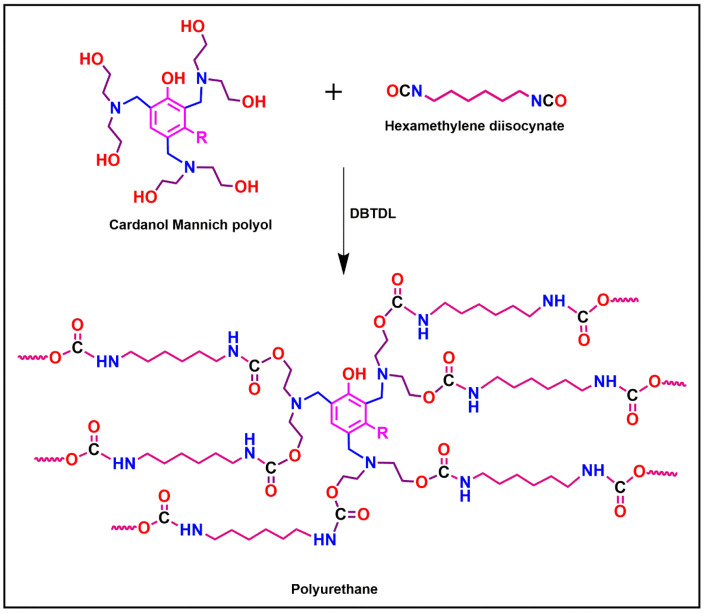
Synthesis of polyurethane from renewable-based cardanol Mannich polyol.

The MS substrate was pre-treated with sandpaper, degreased with acetone, and dried in an oven for 20 min. The required quantities of MHAP of 0, 1, 2, 3, 4, and 5 wt. % and CMP were dispersed into the xylene with stirring. Then, the calculated amounts of hexamethylene diisocyanate (HDI) in the ratio of NCO:OH 1.2:1 and DBTDL as a catalyst were added into the above mixture. After achieving desired viscosity to the resultant formulation, it was applied by brush on pre-treated mild steel (MS) panels of 2.14-inch dimension. The prepared coating panels were allowed to cure at room temperature for 48 h, and the cured coating film’s thickness was 90 ± 2 µm. The prepared samples were coded as CMPU, CMPU-1, CMPU-2 CMPU-3, CMPU-4, and CMPU-5 based on the amount of MHAP. The schematic of PUs preparation reaction is represented in Scheme 2.

## 3. Characterization of Materials

### 3.1. End Group Analysis

Hydroxyl functionality is the most important parameter in the development of polyurethane. They are deciding the actual quantity of diisocyanates for solid film development in the final crosslinked structure of PUs. It was determined experimentally by following ASTM D 6342-12 methods.

### 3.2. Structural Analysis

Transformation of the functional group of cardanol to cardanol Mannich polyol and structural confirmation were determined by recording FT-IR spectra on a Perkin Elmer-1750 in the range between 4000 and 400 cm^−1^ and ^1^H NMR spectroscopic techniques were applied on a Bruker Avance-400 MHz spectrometer in deuterated CDCl_3_ as a solvent and TMS as an internal standard. 

### 3.3. Testing Properties of Coatings

#### 3.3.1. Gloss Test

The lustrous property of PU coatings was investigated using a digital gloss meter (Model BYK Additives and Instruments, Wesel, Germany) at an angle of 60°. For testing, 10 different positions on the surface of coated MS panels were considered and their average value was recorded as a final gloss of PU coatings.

#### 3.3.2. Adhesion Test

The adhesion of developed coatings to the mild steel surface was checked using a cross-cut adhesion tester (model No. 107, Elcometer UK) as per the following method: ASTM D-3359-02. The tester toolbox contained a die of parallel sets of 10 blades, adhesive tape (Scotch brand 810 magic tape), brush, and lens. Initially, the coated surface was rapidly scratched two times at 90° to each other with the help of a blade and smoothly cleaned using a brush. Then, the adhesive tape was pressed on the scratched surface and pulled within 60 S at a 180° angle. Finally, the visual confirmation of the percentage of squares adhered on the surface of adhesive tape from crosscut MS panel with respect to the total initial number of squares was considered for calculating the adhesion of the coatings with the metal substrate.

#### 3.3.3. Pencil Hardness

The hardness of PU-coated samples was determined using a pencil hardness tester (Model BYK Additives and Instruments, Wesel, Germany) as per the ASTM D-3363 standards. The test was carried out by pushing pencils on the coated samples at an angle of 45° and repeated at a fresh place every time until the pencils formed scratches on the surface of coatings.

#### 3.3.4. Flexibility

The flexibility of the prepared coating samples was tested by a Conical Mandrel Instrument (Raj Scientific Co., Mumbai, India) in the range of 45−180° angles.

#### 3.3.5. Contact Angle

The surface hydrophobicity of PU coating samples was determined using a Contact Angle Tensiometer, Model 200 standard Goniometer (p/n 200-F4) of Rame-hart Instrument Co., Succasunna, NJ, USA. The test samples were fitted on the stage of the equipment and a liquid drop of deionized water with constant volume (approximately 3–5 μdm^3^) was dropped on the surface of the PU-coated sample using a microsyringe (Thermo Scientific Gilmont Micrometer Syringe Model-GS-1200). All measurements were taken at room temperature and an average of 10 times was considered for reporting.

#### 3.3.6. Corrosion Performance

The corrosion performance of developed PU coatings was examined by immersion and electrochemical testing.

An immersion study was used to examine the corrosion performance by dipping uncoated and PU-coated MS samples in 3.5% NaCl aqueous solution for 7 days [45]. After testing, the coated samples were compared with the control for a change in gloss, deterioration, cracking, and partial or complete removal of the film from the surface. The result was in the form of captured images of both before and after testing of all samples. 

The corrosion performance of prepared PU coatings was also checked by electrochemical testing. The analysis was performed by an Auto lab PGSTAT30 potentiostat instrument, and all analyses were carried out at room temperature in an aqueous 3.5 wt. % NaCl solution. The analysis comprises three electrodes: platinum wire as a working, calomel and coated MS strips as counter and working electrodes. The area of the coated panels exposed to the test solution was 1 × 0.5 cm^2^ in all cases and the range of testing current potential was kept between −1 and +1 V at a scan rate of 0.01 V/s.

#### 3.3.7. Chemical Resistance

The chemical resistance was checked by dipping coatings into aqueous acid and alkali solutions, water, and xylene as an organic solvent for 7 days [45,46]. The chemical resistance of PU coatings was also checked by the methyl ethyl ketone rub test as per ASTM D-5402. The test was carried out by rubbing the methyl ethyl ketone wet cotton cloth on the surface of coated samples. The maximum number of double rubs at which the coatings were removed from the surface or when 200 rubs were passed was considered as the final result’s value.

#### 3.3.8. Thermal Property

The thermal property of prepared PU films was studied in the range of 40 to 750 °C using a thermogravimetric analyzer (TGA), Perkin Elmer TGA-4000 (Waltham, MA, USA). All PU films were measured at a heating rate of 10 °C per min under an inert nitrogen atmosphere at a flow rate of 20 mL/min.

#### 3.3.9. Surface Morphology

The surface morphology of the prepared PU films was observed under a scanning electron microscope (SEM) (FESEM, S-4800, Hitachi High Technologies Corporation, Tokyo, Japan). The accelerating voltage was in the range of 0.5 to 30 kV and with an emission current at about 10 µA.

## 4. Results and Discussion

Magnetic nanocomposite materials serve multifunctional purposes in the field of catalysis, colloidal photonic crystals, imaging, nanofluids, data storage, defect sensor, optical filters, environmental remediation, and, in particular, in the biomedical industry because of their unique mechanical, thermal, physical, and chemical properties [47]. The research in the field of hydroxyapatite nanocomposites is still in its infancy and requires controlling fabrication processes for advanced applications.

### 4.1. Formation of Cardanol Based polyol

**Figure 1:**
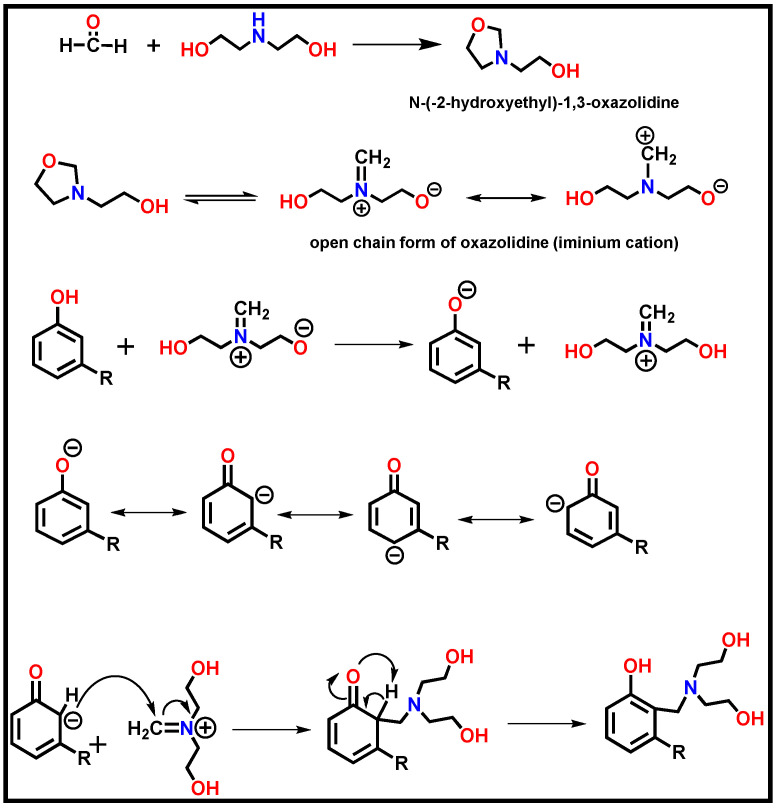
Mechanism for the formation of cardanol Mannich polyol.

Renewable cardanol-based polyol was synthesized by the Mannich condensation of cardanol, diethanolamine, and formaldehyde. The overall process for the synthesis of CMP was considered ecofriendly due to its solvent-less nature. The hydroxyl value of polyol was 187 mg of KOH/g. Initially, diethanolamine and formaldehyde were condensed together to form the oxazolidine intermediate [48]. Oxazolidine is present in the equilibrium with cyclic and open-chain forms. The open-chain form is an iminium cation, which is the well-known intermediate of Mannich reactions [49,50,51]. In the presence of a base (i.e., oxazolidine and tertiary amine), cardanol dissociates in the cardonalate anion with the negative charge circulated at equilibrium in resonance hybrids at oxygen and ortho- and para-positions of the aromatic ring. Finally, it results in a substituted product at ortho- and para-positions of the phenolic hydroxyl group of cardanol. The mechanism for the synthesis of CMP is presented in Figure 1.

**Figure 2:**
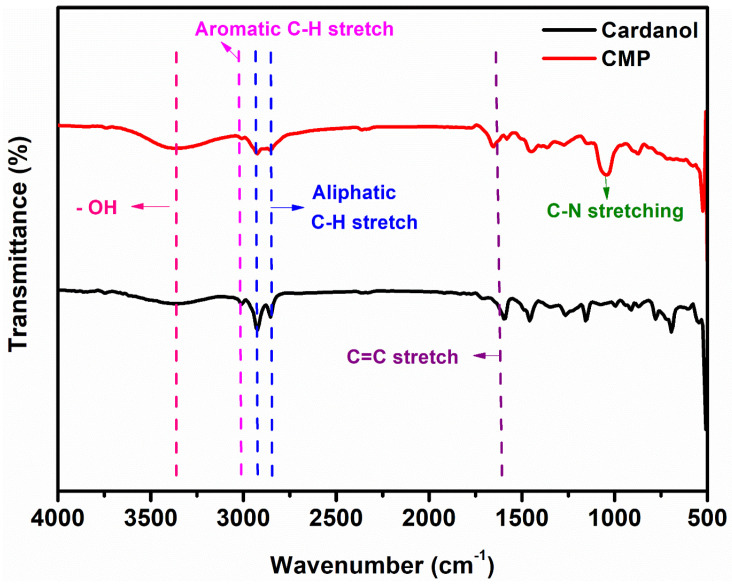
FT-IR spectra of cardanol and cardanol Mannich polyol.

Structural confirmation of the cardanol-based polyol was performed by FT-IR and ^1^H NMR spectroscopies. The FT-IR spectrum of CMP is represented in Figure 2 and the corresponding functional groups are tabulated in Table 2.

The stretching vibration at 3371 cm^−1^ was related to the -OH group present in cardanol and CMP. Absorption bands associated with the vibration of symmetric and asymmetric -CH_2_ group were observed in between 2854 and 2924 cm^−1^, while aromatic -C-H stretching vibration was observed at 3009 cm^−1^. The band at 1651 cm^−1^ is due to the presence of -C=C- bond present in CMP and cardanol. The new broad peak observed in the FT-IR spectra of CMP at 1041 cm^−1^ for -C-N- stretching confirmed that the reaction of oxazolidine with cardanol formed cardanol Mannich polyol.

**Figure 3:**
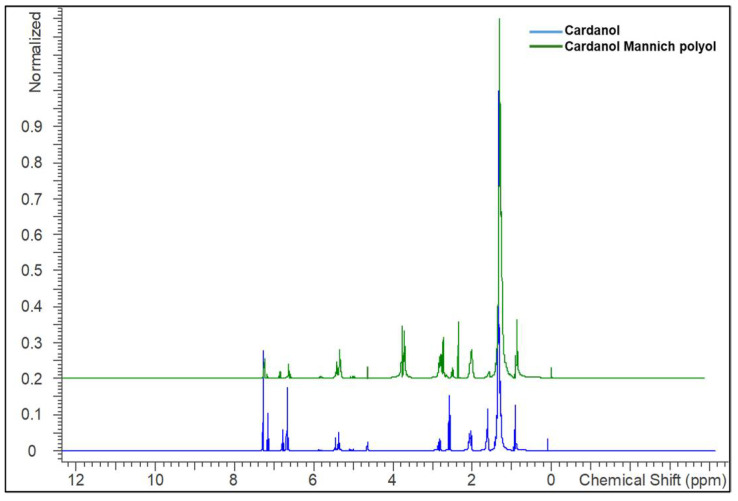
^1^H NMR of cardanol and cardanol Mannich polyol.

Additionally, the structural confirmations of cardanol and CMP were carried by ^1^H NMR, and their spectra are shown in Figure 3. In the spectrum of cardanol, the chemical shift of terminal methylene proton appeared at 0.9 ppm and all -CH_2_- linkages were observed at 1.25–1.5 ppm. The peaks found between 5.03 and 5.46 ppm were related to the protons of -C=C-, while the peaks at 2.03 and 2.55 ppm corresponded to the -CH_2_- protons adjacent to the unsaturation and aromatic ring, respectively. The peak at 4.6 and 6.68–7.17 ppm were related to phenolic protons and aromatic benzene rings. In the spectrum of CMP, the new chemical shift was observed at 2.35 and 3.7 ppm, which were corresponded to the hydroxy and methylene protons present in between the nitrogen atom and phenolic aromatic ring, respectively [49]. The appearance of these protons in the CMP spectrum evidenced the occurence of the reaction between oxazolidine with cardanol.

### 4.2. FT-IR of Magnetic Hydroxyapatite Nanoparticles

**Figure 4:**
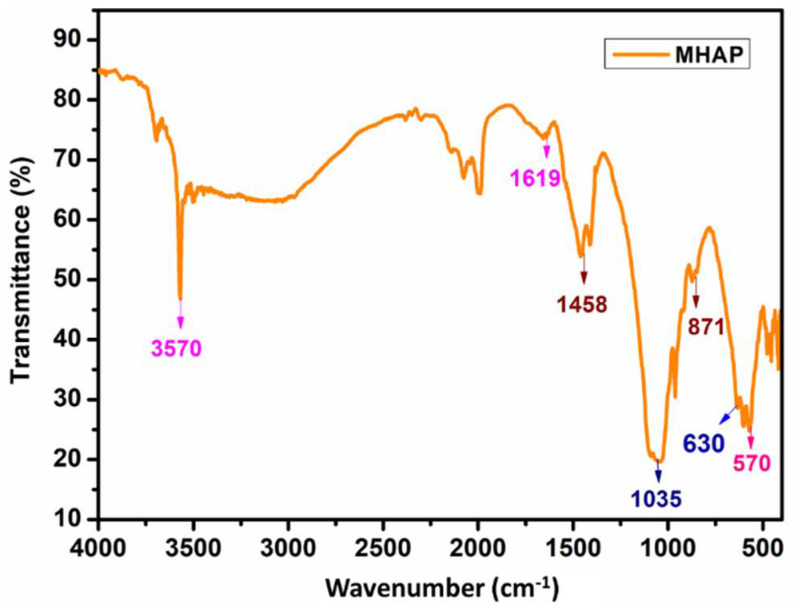
FT-IR spectrum of magnetic hydroxyapatite nanoparticles.

FT-IR spectrum of MHAP nanoparticles is represented in Figure 4, and the values are tabulated in Table 2. The broad absorption band at 3570 cm^−1^ was related to -OH bending vibration. The stretching vibration of carbonyl was observed at 1458, and 871 cm^−1^, while the phosphate group showed absorption at 1035 and 630 cm^−1^ [52,53]. The Fe-O bond stretching vibration occurred at 1619 and 570 cm^−1^ [37]. It proved that MHAP nanoparticles were successfully formed.

### 4.3. Magnetic Behavior of MHAP Nanoparticles

**Figure 5:**
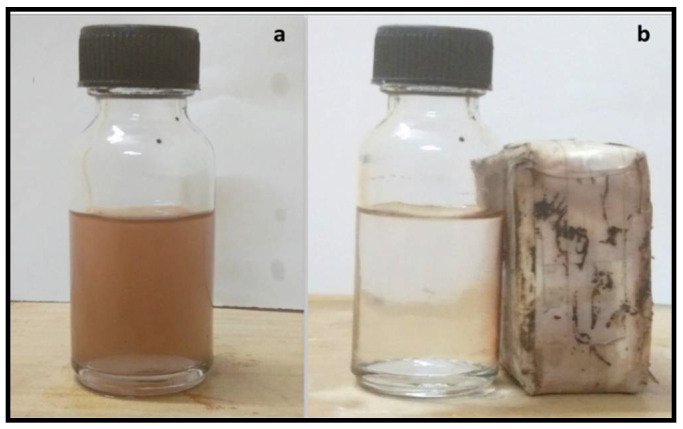
Magnetic behavior of the MHAP nanoparticles (**a**) suspension of nanoparticles and (**b**) MHAP nanoparticles attracted toward the magnet.

The magnetic property of the nanoparticles was tested by a simple magnet test. For this purpose, the synthesized nanoparticles were suspended in a water–ethanol solution and sonicated for 5 min to form a complete suspension of nanoparticles (Figure 5a). Afterward, the magnet was connected to bottles as shown in Figure 5b. In a few seconds, all nanoparticles were attracted toward the magnet, which concluded that the synthesized particles had magnetic behavior.

### 4.4. Coating Properties

Determining the properties of coating is important in order to find out the suitability of coating formulations. The results of coating properties are represented in Table 3. The gloss of all the coated samples was observed in the range of 73 to 121. The gloss value of the pristine sample was more than all nanocomposite coatings because the incorporation of MHAP nanoparticles would have increased opacity as well as increased roughness on the surface of the coating. Thus, as the percentage of MHAP increased in coating formulations, it decreased gloss. All coatings showed 100% adhesion towards the metal surface as not a single block was removed from the area of the scratched surface. Hence, the results conclude that the formulated coatings have excellent adhesion to the metal surface. The MHAP nanoparticles incorporated coatings were not scratched to the level of a 5H-grade pencil. The coatings containing 4 and 5% MHAP nanoparticles presented better pencil hardness (5 H) than other coating samples. It may be due to the magnetic nature of MHAP nanoparticles that might have resulted in high interactive forces between metal surfaces and the coating matrix. All coating samples passed the flexibility test, which can offer the prevention of crack formation. In addition to that, the chemical resistance of all the prepared coatings was checked using the methyl ethyl ketone rub test. There were no defects found on the surface of coatings, such as film removed from the surface, crack formation, and changing color of coating on the surface up to 200 double rubs. Finally, based on all coating properties, one can say that the developed coatings are suitable for the coatings on metal surfaces.

### 4.5. Chemical Resistance Study

**Figure 6:**
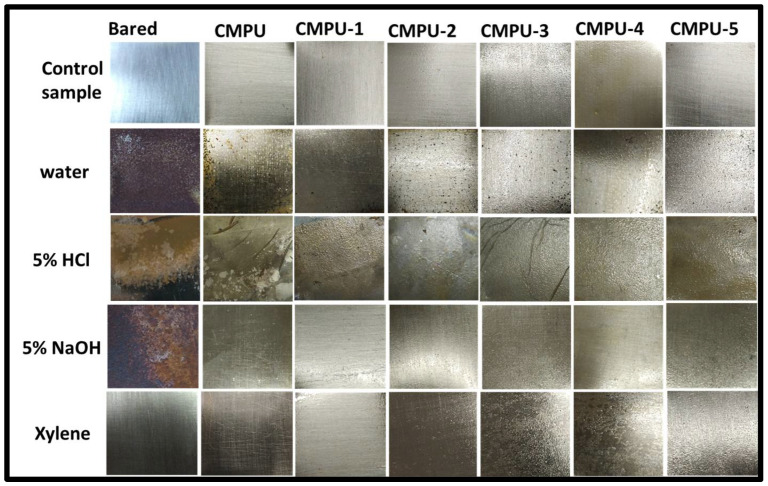
Chemical resistance of bared and prepared PU coatings.

The chemical resistance of the developed cardanol-based nanocomposite coatings was studied in 5% HCl (in DI water) and 5% NaOH (in DI water), solutions, water, and xylene as an organic solvent for 7 days. The obtained results of the test are expressed in Table 4 and captured images are provided in Figure 6. 

The results detected that bared and MHAP nanoparticles added PU coating samples were completely damaged, had film detached from the surface, and showed a loss in gloss in water and acid media. On the other hand, PUs added with MHAP showed better resistance against media, with the exception of only a slight loss in gloss. These results indicated that the presence of MHAP plays a role in increasing the adhesion of metal surfaces. A minor loss in gloss was noted for all coatings in the alkali medium, while all prepared coatings showed excellent results against the solvent medium, which may be attributed to the good interaction between the MHAP and polyurethane matrix. Thus, all composite coatings with MHAP showed better chemical resistance compared to the pristine PU.

### 4.6. Anticorrosive Performance by Immersion Method

**Figure 7:**
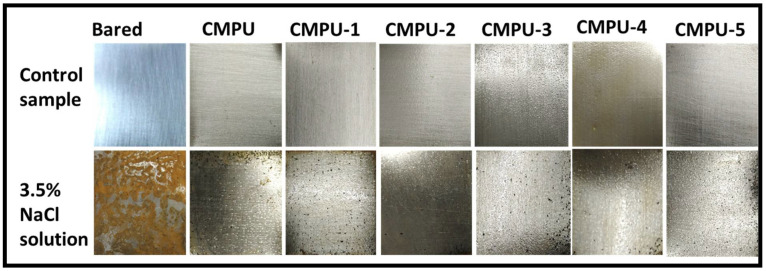
Anticorrosive performance of bared and prepared PU coatings.

The corrosion resistance of the prepared nanocomposite coatings was examined by dipping coated and uncoated samples in 3.5% NaCl solution. After the test, analyzed samples were compared with control samples and captured images are given in Figure 7. The bared sample fully corroded, as it does not cover the PU coating layer and had direct contact with the corrosive medium. From the test results, it was revealed that MHAP-based nanocomposite coatings provide superior corrosion resistance than compared to the bared and without MHAP coatings. The presence of MHAP nanoparticles in the PU matrix provides a strong adhesion over the metal surface and acts as a barrier between corrosive media and metal surface, which caused inhibition in the corrosive process.

### 4.7. Anticorrosive Study by Electrochemical Method

**Figure 8:**
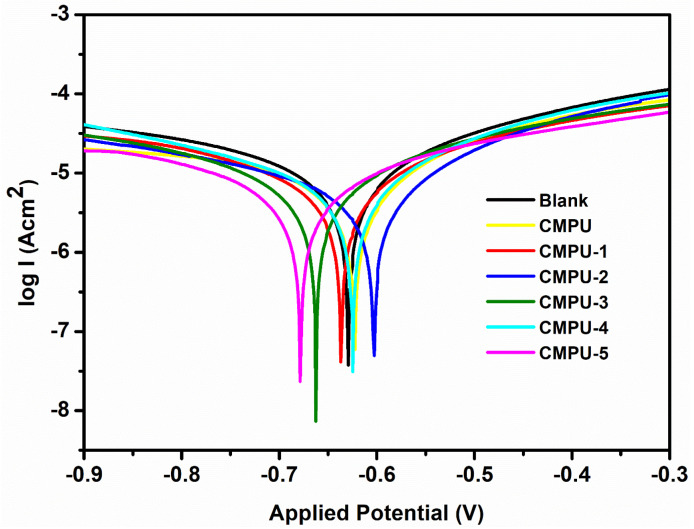
Tafel plot of CMPU, CMPU-1, CMPU-2, CMPU-3, CMPU-4, and CMPU-5 coated samples in aq. 3.5% NaCl medium.

Anticorrosive performance was also examined by measuring Tafel plots of uncoated, coated, and MHAP-added coating samples in 3.5% NaCl solution. The Tafel plots of all PU samples are shown in Figure 8. The plots were used to estimate corrosion potential (E*_corr_*), corrosion current density (I*_corr_*), polarization resistance (Rp), and corrosion rate (CR). Using the Tafel extrapolation method based on the software Nova 1.8, the values of these corrosion parameters were calculated and represented in Table 5. In such a curve, higher E*_corr_* and lower I*_corr_* values corresponded to the lower corrosion rate and vice versa [6,54]. The MHAP nanoparticles-added coatings showed lower I*_corr_* values and higher E*_corr_* values than blank and pristine (CMPU) coatings. As the percent loading of MHAP in formulations increased, it decreased the I*_corr_* values of the coatings because of an increase in adherence of the coatings with the metal surface due to the magnetic property of MHAP particles and the formation of a protective barrier between corrosive ions and metal substrate.

**Figure 9:**
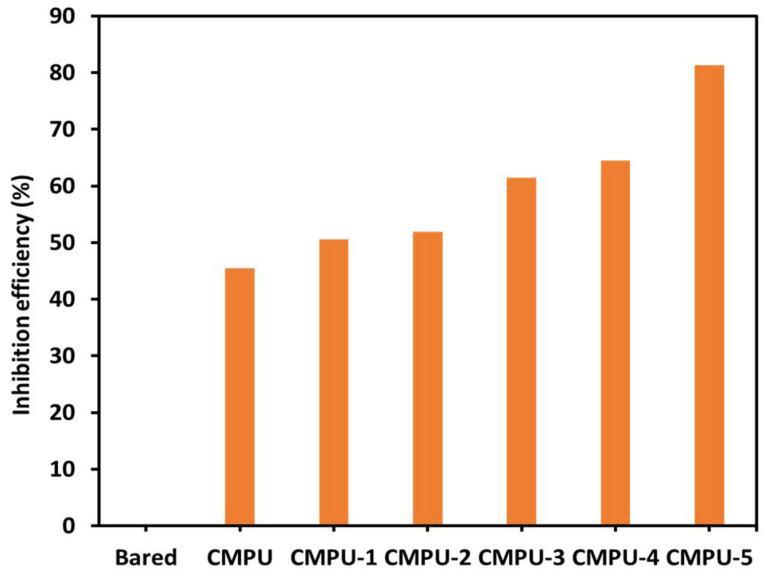
The inhibition efficiency of bare, CMPU, CMPU-1, CMPU-2, CMPU-3, CMPU-4, and CMPU-5 coated samples tested against aq. 3.5% NaCl solution.

Percent IE was determined from the values of corrosion current density of uncoated and coated samples. In general, the higher the I*_corr_* values, the lower the inhibition efficiency of the coatings. The graphical presentation of all the coated and uncoated samples is shown in Figure 9. The corrosion inhibition efficiency of MHAP-added coating samples was far better than blank and CMPU. Higher efficiency was obtained for the 5% loaded coating due to the good adhesion of PU formulation to the MS substrate. Additionally, the hydrophobic nature of the coatings might have helped to enhance anticorrosive behavior by restricting interaction between coatings and corrosive media.

**Figure 10:**
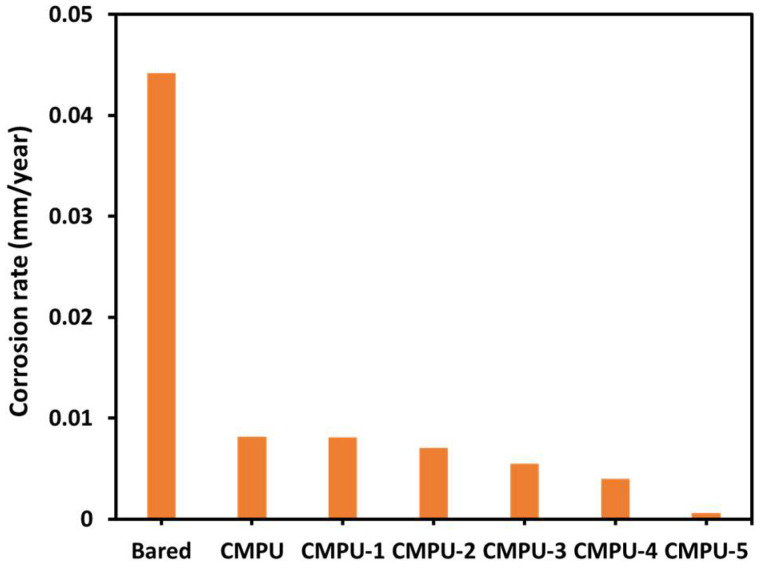
Corrosion rate of bare, CMPU, CMPU-1, CMPU-2, CMPU-3, CMPU-4, and CMPU-5 coated samples tested against aq. 3.5% NaCl medium.

The corrosion rate versus the type of coatings is graphically represented in Figure 10. The corrosion rate of all PU-coated samples was better than the uncoated ones. Therefore, it confirmed that the prepared PU formulations acted as an obstacle for said medium to interact with the substrate and thus decreased the corrosion rate. From all results, it was concluded that the developed nano-composite PU coatings provide superior corrosion inhibition as compared to CMPU due to the presence of MHAP nano-particles in the PU matrix. MHAP nano-particles act as a barrier to corrosive ions, which cause corrosion inhibition and result in high Rp values and impedance of nanocomposite coatings, which increased with an increase in the percent loading of MHAP nanoparticles in coatings. 

### 4.8. Contact Angle

**Figure 11:**
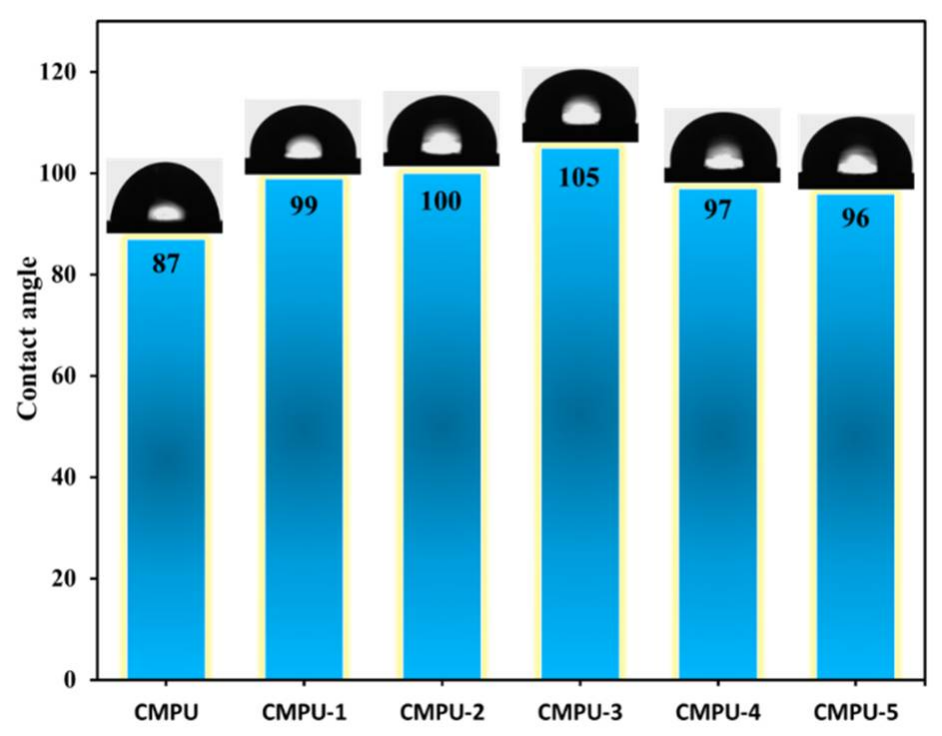
Contact angle of CMPU, CMPU-1, CMPU-2, CMPU-3, CMPU-4, and CMPU-5 coated samples.

The contact angle of the coated CMPU and all MHAP-embedded coated samples was measured to estimate the surface hydrophobicity, and the results are shown in Figure 11. From the results, it was observed that all nanocomposite-coated MS samples were more hydrophobic than the pristine-coated sample. The contact angle of all the nanocomposite coatings was found to be more than 90°, which was much higher compared to MHAP coatings (87°) without nanoparticles. The contact angle of the coating samples increased with an increase in the amount of MHAP nanoparticles up to 3% and, beyond that, the declining values of the contact angle were observed. Overall, the results of contact angle revealed the hydrophobic nature of the prepared nanocomposite PU coatings. 

### 4.9. Thermogravimetric Analysis

**Figure 12:**
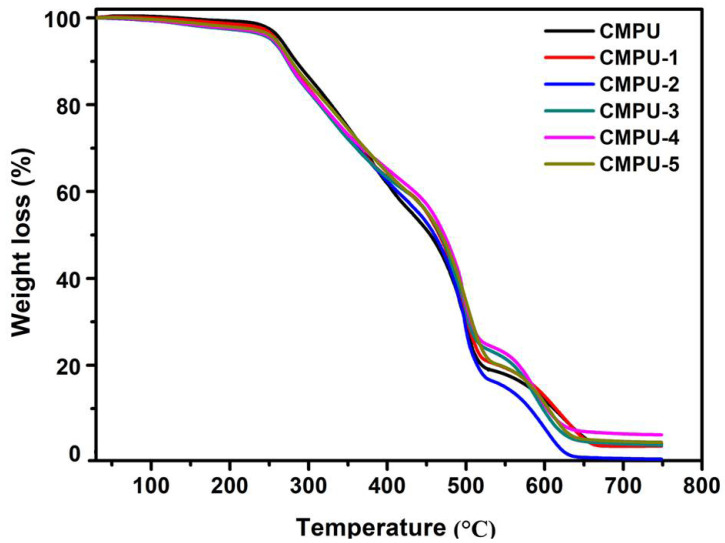
Thermogravimetric curves of CMPU, CMPU-1, CMPU-2, CMPU-3, CMPU-4, and CMPU-5 coated sample.

The thermograms of all prepared PU films are presented in Figure 12. Thermal analysis showed three steps of thermal degradation in all PU films. The first step of degradation started in the range of 212–225 °C and ended at 410–419 °C with a degradation result of 30–38% weight losses due to the breakdown of urethane groups in PUs. In the second step, degradation occurred in the range of 416–425 °C and ended at 530–539 °C with weight losses of 35–43% due to the degradation of the main backbone chain of PUs. The third step of degradation was observed in the range of 534–536 °C and completed at 643–677 °C with weight losses of 17–19% as a result of residual degradation. Thus, it can be stated that all prepared composite coatings resulted in excellent thermal stability than CMPU.

### 4.10. Surface Morphology of MHAP Composite Coating

**Figure 13:**
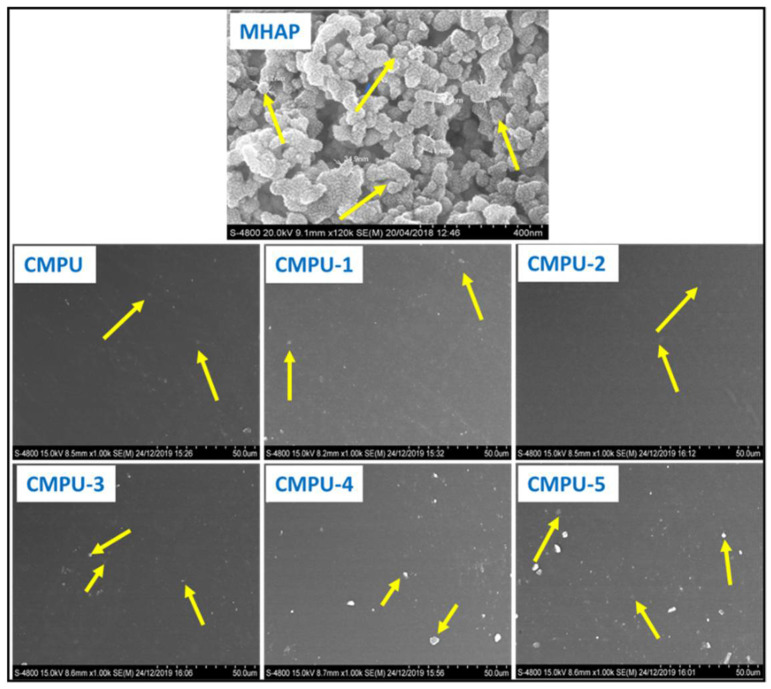
SEM images of MHAP, CMPU, CMPU-1, CMPU-2, CMPU-3, CMPU-4, and CMPU-5 coated samples.

The MHAP nanoparticles and their PU coatings were observed under a scanning electron microscope, and a few representative images are shown in Figure 13. The morphology of MHAP nanoparticles revealed irregularly shaped agglomerates with an average size of 38.64 nm. The particles showed a smooth surface. The CMPU appeared as a smooth surface as it does not contain MHAP and it was free from phase separation or the presence of any voids. However, slight agglomerations were seen at larger amounts of nanoparticles, CMPU-4 and 5 samples, which is common in the dispersion of nanoparticles. The images of MHAP incorporated coatings were also clear and homogeneous with the absence of any type of phase separation or cracks over the surface. Therefore, it can be concluded that MHAP was properly dispersed in the PU formulation and interacted with the matrix. Furthermore, all coatings were free from the microcracks, voids, and phase separation.

## 5. Conclusions

Cardanol was used as a renewable phenol for the preparation of Mannich-type polyol, which was further utilized in the formulation of PU nanocomposite coatings using a various percentage of MHAP (1,2,3,4, and 5%). The structural features of the synthesized cardanol Mannich polyol were confirmed by FT-IR and ^1^H NMR spectroscopic techniques. MHAP was synthesized in the laboratory and characterized by FT-IR and SEM analysis. The developed PU nanocomposite coatings demonstrated good physicochemical properties. The prepared coatings showed excellent anti-corrosion and chemical resistance tests. The hydrophobic character of coatings increased up to 3% loading of MHAP; beyond that, it decreased as measured by the contact angle test. All coatings showed good thermal stability and smooth surface morphology, as studied by TGA and SEM, respectively. Thus, cardanol can be used as a good renewable substitute over petroleum materials in the preparation of polyurethane coatings.

## Figures and Tables

**Table 1:** The elemental composition of mild steel substrate used for exploring the functionality of the coating.

Element Composition	Amount in Steel (%)
Carbon	0.16
Aluminum	0.07
Silicon	0.168
Manganese	0.18
Phosphorous	0.025
Copper	0.09
Iron	Balance

**Table 2:** Peaks observed in FT-IR spectra of cardanol and cardanol Mannich polyol and magnetic hydroxyapatite nanoparticles and the corresponding functional groups.

Wavenumber (cm^−1^)	Vibration Type
Cardanol and cardanol Mannich polyol	
3371	-OH bending vibration
2854–2924	vibration of symmetric and asymmetric -CH_2_ group
3009	-C-H stretching vibration
1651	C=C-bond
1041	-C-N- stretching
Magnetic hydroxyapatite nanoparticles	
3570	-OH bending vibration
1458, and 871	-C=O stretching vibration
1035 and 630	phosphate group
1619 and 570	Fe-O bond stretching

**Table 3:** Coating properties of prepared PUs coatings.

PUs Code	Gloss 60°	Cross-Cut Adhesion (%)	Pencil Hardness	Flexibility	Methyl Ethyl Ketone Double Rub Test
CMPU	121	100	3H	Pass	200
CMPU-1	96	100	3H	Pass	200
CMPU-2	85	100	4H	Pass	200
CMPU-3	84	100	4H	Pass	200
CMPU-4	74	100	5H	Pass	200
CMPU-5	73	100	5H	Pass	200

**Table 4:** Chemical resistance of bared, CMPU, CMPU-1, CMPU-2, CMPU-3, CMPU-4, and CMPU-5.

Sample Code	Water	HCl	NaOH	Xylene
Bared	F	F	F	F
CMPU	B	D	A	A
CMPU-1	B	B	A	A
CMPU-2	C	C	A	A
CMPU-3	B	C	A	A
CMPU-4	B	B	A	A
CMPU-5	B	B	A	A

A = not affected; B = slight loss in gloss; C = change in color and loss in gloss; D = the film partly removed; E = film completely removed; F = fully damaged.

**Table 5:** Electrochemical corrosion measurement of bare, CMPU, CMPU-1, CMPU-2, CMPU-3, CMPU-4, and CMPU-5 coated samples.

Sample Code	E*_corr_* (mV)	I*_corr_* (nA)	Rp (kΩ)	Corrosion Rate (CR) (mm/Year)	Inhibition Efficiency (% IE)
Bare (Uncoated)	−656.47	968.44	4.929	0.0442	0.00
CMPU	−618.24	528.12	8.994	0.00814	45.47
CMPU-1	−602.31	479.09	8.954	0.00812	50.52
CMPU-2	−544.68	465.71	10.343	0.00709	51.91
CMPU-3	−554.76	372.97	23.280	0.005483	61.48
CMPU-4	−547.84	345.10	16.059	0.00401	64.47
CMPU-5	−535.07	181.35	116.520	0.000582	81.28

## Data Availability

Not applicable.
